# Management of Ventilator-Associated Pneumonia: Quality Assessment of Clinical Practice Guidelines and Variations in Recommendations on Drug Therapy for Prevention and Treatment

**DOI:** 10.3389/fphar.2022.903378

**Published:** 2022-05-20

**Authors:** Hong-Yan Li, Hai-Shan Wang, Ying-Lin Wang, Jing Wang, Xue-Chen Huo, Quan Zhao

**Affiliations:** ^1^ Department of Pharmacy, Qindao University Medical College Affiliated Yantai Yuhuangding Hospital, Yantai, China; ^2^ Department of Intensive Care Unit, Yantai YEDA Hospital, Yantai, China; ^3^ Department of Hepatobiliary Surgery, Qindao University Medical College Affiliated Yantai Yuhuangding Hospital, Yantai, China

**Keywords:** ventilator-associated pneumonia, drug prevention and treatment, clinical practice guideline, AGREE II, recommendation

## Abstract

**Purpose:** To assess the quality of clinical practice guidelines (CPGs) related to drug therapy for prevention and control of ventilator-associated pneumonia (VAP) and compare the differences and similarities between recommendations.

**Methods:** Electronic databases (including PubMed, Cochrane library, Embase, Web of Science), guideline development organizations, and professional societies were searched to identify CPGs for VAP from 20 January 2012 to 20 January 2022. The Appraisal of Guidelines Research & Evaluation (AGREE) II instrument was used to evaluate the quality of the guidelines. The recommendations on drug therapy for prevention and treatment for each guideline were extracted, and then a descriptive synthesis was performed to analyze the scope/topic, and consistency of the recommendations.

**Results:** Thirteen CPGs were included. The median score and interquartile range (IQR) in each domain are shown below: scope and purpose 72.22% (63.89%,83.33%); stakeholder involvement 44.44% (38.89%,52.78%); rigor of development 43.75% (31.25%,57.29%); clarity and presentation 94.44% (77.78%,94.44%); applicability 20.83 (8.34%,33.34%) and editorial independence 50% (33.33%,66.67%). We extracted 21 recommendations on drug therapy for prevention of VAP and 51 recommendations on drugs used for treatment. Some controversies remained among the included guidelines.

**Conclusion:** There is considerable variability in the development processes and reporting of VAP guidelines. Despite many similarities, the recommendations still had some inconsistencies in the details. For the prevention and treatment of VAP, local microbial epidemiology and antibiotic sensitivity must be considered, and recommendations should be regularly revised as new evidence emerges.

## 1 Introduction

### 1.1 A Basic Introduction to Ventilator Associated Pneumonia

Ventilator-associated pneumonia (VAP) is a special type of nosocomial infection typified by pulmonary parenchymal inflammation, which usually occurs 48 h after artificial airway or mechanical ventilation (Infectious disease group RmboCMA 2018). VAP is believed to be an important cause of healthcare-associated infections, resulting in increased morbidity and mortality, it is one of the most frequently occurring infections in the intensive care unit (ICU) (Sangale et al., 2021). Despite the rapid development of critical care medicine, the incidence rate and mortality of VAP remain high. VAP is reported to affect 5–40% of patients receiving invasive mechanical ventilation for more than 2 days, with large variations depending upon the country, ICU type, and criteria used to identify VAP (American Thoracic 2005; Seguin et al., 2014). The data from the International Nosocomial Infection Control Consortium (INICC) confirmed that the incidence of VAP was 14.1/1000 mechanical ventilator-days, and the mortality was 36.6% (Rosenthal et al., 2020). VAP can also prolong hospitalization and intubation times, increase the use of antibiotics, affect the prognosis of severely ill patients, and increase medical expenses (Kollef et al., 2012; Álvarez-Lerma and Sánchez García 2018; Papazian et al., 2020). Therefore, curbing VAP has become the most urgent problem facing medical institutions. Microbiological tools have currently made progress, but the epidemiology and diagnostic criteria of VAP are still controversial, which complicates the interpretation of prevention, treatment, and outcome research (Nair and Niederman 2015; Timsit et al., 2017).

### 1.2 Antimicrobial Resistance

Antimicrobial resistance is not only a global crisis, but also a global problem occupying the attention of both governments and society. The Antibiotic Resistance Global Report on Surveillance issued by the World Health Organization (WHO) in April 2014 (WHO 2014). It is reported that in the Americas, *Escherichia coli* has high resistance to the third generation cephalosporins and fluoroquinolones, and *Klebsiella pneumoniae* has strong and widespread resistance to the third generation cephalosporins, Methicillin resistant *Staphylococcus aureus* (MRSA) was present in up to 90% of patients in some parts of the region. In Europe, *Klebsiella pneumoniae* is highly resistant to the third generation cephalosporins, MRSA was present in up to 60% of patients in some parts of the region (WHO 2014). At present, in China, the overall prevalence of MRSA remains at about 35%, the proportion of *Escherichia coli* resistant to third-generation cephalosporins is still more than 55%, the proportion of *Pseudomonas aeruginosa* resistant to carbapenems remains around 20%, and the proportion of *Acinetobacter baumannii* resistant to carbapenems is on the rise, at nearly 60% (Zhang et al., 2016). In 2001, WHO published the “WHO global strategy for containment of antibiotic resistance”, to address the problem of bacterial drug resistance. This put forward global action suggestions to deal with antibiotic resistance (WHO 2001). The UK announced its “5-year antibiotic resistance strategy 2013 to 2018” in 2013 (Affairs DoHfEFaR 2013). In 2016, China issued the “national action plan to curb bacterial drug resistance (2016–2020)”, requiring all large medical institutions to attach great importance to the clinical application of antibiotics and improve their management strategies (Ministry of education 2016).

At the same time, countries have implemented clinical guidelines to further standardize the medication use by professionals and the public. The National Institute for health and Care Excellence (NICE), a British Government institution, has formulated evidence-based clinical medication guidelines for antibiotics to guide the rational use of antibiotics and increase the clinical management of antibiotic use. At the same time, the Advisory Committee on Antimicrobial Resistance and Healthcare Associated Infection (ARHAI) in British released the clinical guidelines with the theme of “Start Smart-then Focus” (Ashiru-Oredope et al., 2012). China has issued regulations and normative documents such as the “Administrative Measures for the Clinical Application of Antibiotics” to guide the use of antibiotics, but it is necessary to clarify the relevant supervision needed to ensure that the guidelines do play a normative and guiding role (Author Anonymous 2013).

### 1.3 Objective of the Study

Clinical practice guidelines (CPGs) are systematically constructed recommendations formulated to aid decision-making among medical professionals, which provide evidence-based recommendations for clinical practitioners and other healthcare professionals about the management of patients with diseases or other clinical conditions (Rosenfeld et al., 2013; Vandvik et al., 2013). They help to improve the quality of medical treatment and patients’ prognosis (Woolf et al., 1999). To standardize the prevention, diagnosis, and treatment of VAP, many national and international organizations have developed the relevant CPGs. The prevention and treatment of VAP in different countries is based on its pathogenic characteristics and antimicrobial sensitivity, which is significant and important for guiding empirical treatment (Torres et al., 2017; Chou et al., 2018; Infectious disease group RmboCMA 2018; Leone et al., 2018).

To date, there is still uncertainty regarding VAP management (Nair and Niederman 2015). Many CPGs have been developed by different organizations to change the empirical management of VAP. Increased production of CPGs is accompanied by growing concern about variations in quality and recommendations. External validation and prospective evaluation of guidelines are therefore necessary. So we have performed a comprehensive review of guidelines related to drug therapy for prevention and treatment of VAP to assess their methodological quality using the Appraisal of Guidelines for Research & Evaluation (AGREE) II (Ma et al., 2020) instrument and compared the differences between them, to provide a reference for the prevention and treatment of VAP and further promote rational drug use.

## 2 Materials and Methods

### 2.1 Guideline Identification

Relevant guidelines were identified through computerized searches of PubMed, Cochrane library, Embase, Web of Science using a combination of text free terms and their corresponding Mesh terms, as well as three major Chinese academic databases. The search strategy is showed in Supplementary File S1. The important professional society websites regarding critical care medicine and infection were also searched for VAP guidelines, Supplementary File S2 lists the important websites with potential VAP guidelines. In addition, we checked the references of included guidelines and consulted experts in the field.

All guidelines related to drug therapy for prevention or treatment of VAP published in English or Chinese from 20 January 2012 to 20 January 2022 were included. Documents were considered guidelines if they met the following criteria: (Infectious disease group RmboCMA, 2018): A guideline should have a clear recommendation on drug therapy for prevention or treatment of VAP for adults and contain all related supporting materials and documents. (Sangale et al., 2021). Evidence-based guidelines. The guidelines report on search strategies, literature quality or data extraction, and classify the level of evidence (LOE) and the strength of recommendation (SOR). (Seguin et al., 2014). If the guidelines had updated versions, only the most recent version was included.

Exclusion criteria: Single-author overviews, editorials, letters to the editor, textbook-like publications, short summaries, documents without clear recommendations, and secondary publications (including versions translated from other languages) were excluded. If a guideline only applied to children, patients with immunodeficiency or COVID-19, it was also excluded.

### 2.2 Quality Assessment

CPGs were evaluated independently by four assessors from different backgrounds, including one ICU expert (H-SW), two pharmacists (H.-YL, JW), and one methodologist (X-CH). All assessors have extensive experience in evaluating CPGs using the AGREE II instrument. AGREE II consists of 23 key items organized into six domains (Ma et al., 2020). The scope and purpose domain includes the main objectives of the CPG, the target population and health questions; the stakeholder involvement domain concerns the extent to which the CPG was developed by the appropriate stakeholders and represents the opinions of its intended users; the rigor of development domain focuses on the procedure for synthesizing and gathering evidence and the methods used to formulate the recommendations; the clarity of presentation domain focuses on whether recommendations are specific and clear, different options for addressing the condition or health issue are clearly presented, and key recommendations are easily identifiable; the applicability domain assesses processes related to guideline dissemination and implementation, such as additional materials, organizational facilitators and barriers, monitoring or audit and cost implications; the editorial independence domain is concerned with whether the interests or views of the funding body have influenced the forming of the final recommendations and whether the competing interests of all guideline developers have been recorded, addressed and reported. The score for each domain is obtained by summing up all the scores of the individual items in one domain and then standardizing using the following formula: (obtained score - minimum possible score)/(maximum possible score - minimum possible score). The standardized scores ranged from 0 to 100%, a score of 60% average was chosen to establish the proportion of guidelines that scored points above this level in every domain.

### 2.3 Data Collection

We developed a draft data extraction form which included document characteristics (e.g., year of publication, country/region, version, development organization and team) and methodological features of the guideline (e.g., multidisciplinary cooperation, sources of evidence, criteria for selecting the evidence, grading method, methodology used to formulate the recommendations, stakeholder involvement, funding, and disclosure of conflicts of interest). Consistent with the scope of this study, we also tabulated the information on drug therapy for prevention and treatment of VAP, which we used to determine if the recommendations only applied to VAP, as is the case of some recommendations, or if they were pertinent to VAP, however, all evidence supporting the recommendations came from VAP-related research.

### 2.4 Data Statistical Analysis

A descriptive analysis was performed by calculating each domain score and scaled domain score. Agreement among the four assessors was calculated by the intraclass correlation coefficient (ICC) with 95% confidence interval (CI) for each domain. According to the scale proposed by Fleiss, the degree of agreement between 0.00 and 0.40 was deemed poor, 0.41 to 0.75 was fair to good, and 0.75 to 1.00 was excellent (Everitt and Fleiss 1981). Statistical analyses were conducted using SPSS 23.0.

## 3 Results

### 3.1 Search Results and Baseline Characteristics

**FIGURE 1:**
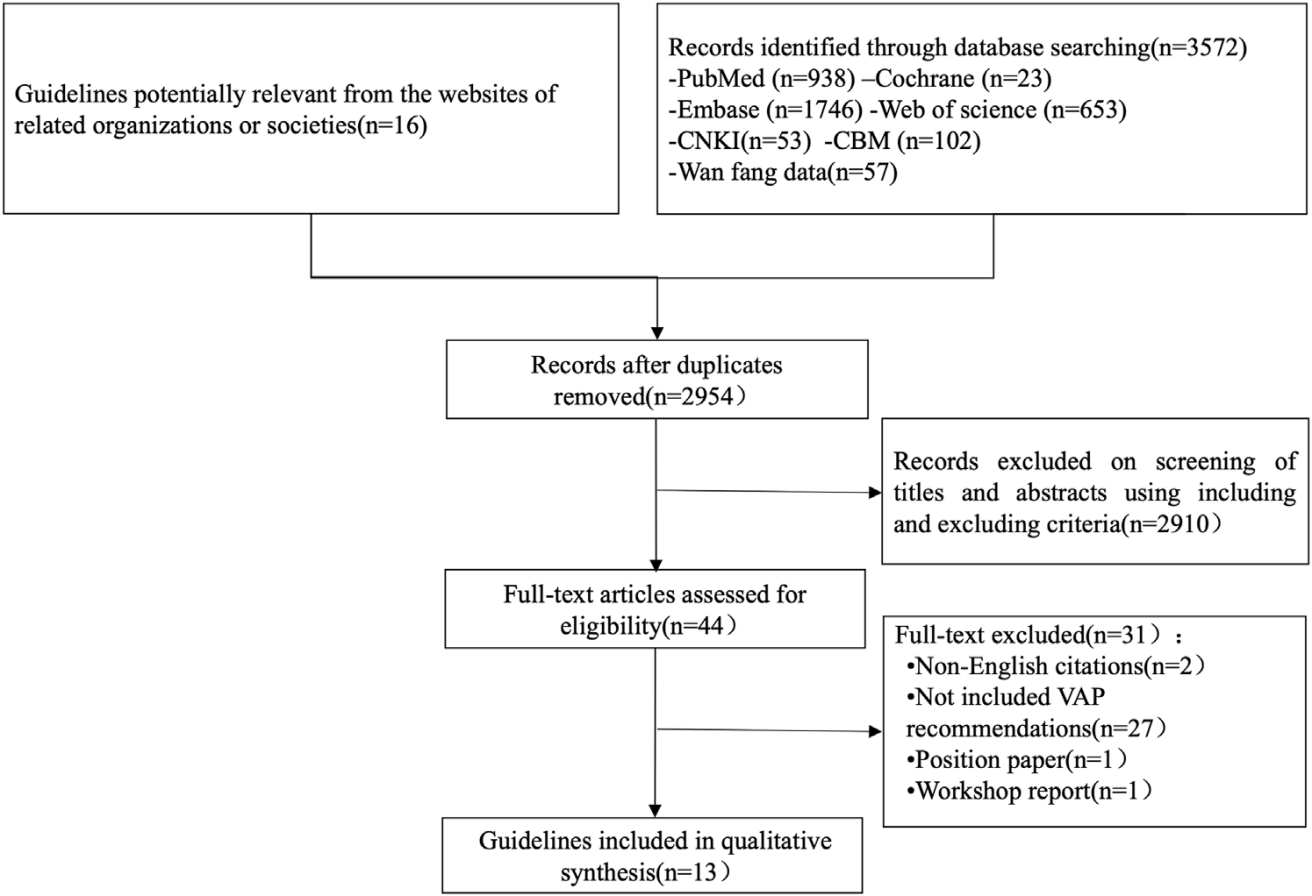
Flow chart of the identification process of CPGs for VAP.

A total of 3572 relevant documents were obtained in the initial examination, and 13 guidelines were finally included according to the inclusion and exclusion criteria (Gupta et al., 2012; Association CcmboCM 2013; Klompas et al., 2014; Mehta et al., 2014; Álvarez Lerma et al., 2014; Kalil et al., 2016; Mikasa et al., 2016; Torres et al., 2017; Chou et al., 2018; Infectious disease group RmboCMA 2018; Leone et al., 2018; Collins et al., 2020; Association SoCRDoCM 2021). The flow chart is shown in Figure 1.

For each guideline finally included, we systematically collected all relevant information and data. All guidelines were evidence-based. Five guidelines were updated versions (Klompas et al., 2014; Kalil et al., 2016; Mikasa et al., 2016; Chou et al., 2018; Infectious disease group RmboCMA 2018). The general characteristics of the included guidelines are listed in Table 1.

**TABLE 1:** General characteristics of the included guidelines.

Guideline	Country	Developing Organization	Target Population	Theme of Recommendations	Version
Qiu, HB 2021 (Association SoCRDoCM, 2021)	China	SCRD of CMA	Patients with mechanical ventilation	VAP treatment	First Version
Collins, T. 2020 (Collins et al., 2020)	British	BACCN	Critically ill adult patients	VAP prevention	First Version
Chou, C.C. 2018 (Chou et al., 2018)	Taiwan, China	IDST/TSPCCM	CAP, HAP, VAP, HCAP in adults and pediatric pneumonia	VAP prevention	Updated
				VAP treatment	
Qu, JM 2018 (1)	China	IDG of RMBCMA	Non-immunocompromised patients with HAP/VAP over 18	VAP prevention	Updated
				VAP treatment	
Lenoe, M. 2018 (Leone et al., 2018)	France	SFAR/SRLF	HAP/VAP (including COPD, neutropenia, post-operative, and pediatrics)	VAP treatment	First Version
Torres, A. 2017 (Torres et al., 2017)	Europe	ERS/ESICM/ESCMID/ALAT	Adult patients with HAP and VAP, does not apply to patients with primary and secondary immune deficiency	VAP prevention	First Version
				VAP treatment	
Mikasa, K. 2016 (Mikasa et al., 2016)	Japan	JAID/JSC	Patients with respiratory infectious diseases in Japan and covered all such diseases in adults and children	VAP treatment	Updated
Kalil, A.C. 2016 (Kalil et al., 2016)	America	IDSA/ATS	Non-immunocompromised patients with HAP/VAP	VAP treatment	Updated
Mehta, Y. 2014 (Mehta et al., 2014)	India	ISCCM	Patients at risk of nosocomial infections	VAP prevention	First Version
Klompas, M. 2014 (Klompas et al., 2014)	America	SHEA/IDSA/AHA/APIC	VAP	VAP prevention	Updated
Alvarez-Lerma, F. 2014 (Álvarez Lerma et al., 2014)	Spain	SSICM/SSICN	VAP	VAP prevention	First version
Li, YM 2013 (Association CcmboCM, 2013)	China	CCMCMA	VAP	VAP prevention	First Version
				VAP treatment	
Gupta, D. 2012 (Gupta et al., 2012)	India	ICS and NCCP	VAP/HAP in adults	VAP prevention	First Version
				VAP treatment	

HAP: Hospital-acquired Pneumonia; VAP: Ventilator-associated Pneumonia; SCRD, of CMA: Subgroup of Critical Respiratory Diseases of Chinese Medical Association; BACCN: British Association of Critical Care Nurses; ISCCM: Indian Society of Critical Care Medicine; IDST: Infectious Diseases Society of Taiwan; TSPCCM: Taiwan Society of Pulmonary and Critical Care Medicine; IDG, of RMBCMA: Infectious disease group, Respiratory medicine branch of Chinese Medical Association; SFAR: French Society of Anesthesia and Intensive Care Medicine; SRLF: French Society of Intensive Care; ERS: European Respiratory Society; ERSESICM: European Society of Intensive Care Medicine; ESCMID: European Society of Clinical Microbiology and Infectious Diseases; ALAT: Latin American Thoracic Association; ICU: Intensive Care Unit; COPD: Chronic obstructive Pulmonary Disease; JAID: Japanese Association for Infectious Diseases; JSC: Japanese Society of Chemotherapy; IDSA: Infectious Diseases Society of America; ATS: American Thoracic Society; HAIs: Hospital-acquired Infections; SSICM: The Spanish Societies of Intensive Care Medicine; SSICN: ISCCM: The Spanish Societies of Intensive Care Nurses Indian Society of Critical Care Medicine; AHA: American Hospital Association; APICA: association for Professionals in Infection Control and Epidemiology; CCMCMA: Critical care medicine branch of Chinese Medical Association. ICS, and NCCP: Indian Chest Society and National College of Chest Physicians.

### 3.2 Quality Assessment

**FIGURE 2:**
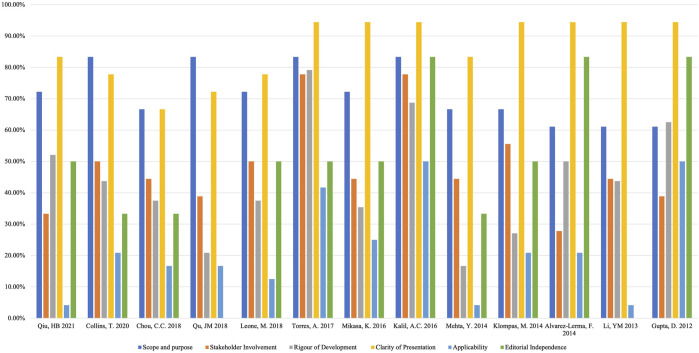
AGREE II Domain scores for included guidelines.

Four assessors independently assessed the 13 guidelines with an ICC value of 0.82 (95% CI = 0.73–0.87), which indicated a high level of reliability among assessors. The quality of the guidelines varied greatly, from fulfilling most of the AGREE criteria to only fulfilling an unsatisfactory number of items. Across all guidelines, none of them had high scores for all domains, and the assessors assigned the highest score to the domain of “clarity of presentation” and the lowest score to “applicability”. The ERS 2017 guideline (Torres et al., 2017) ranked highest in overall quality, whereas the CMA 2018 guideline (Infectious disease group RmboCMA 2018) ranked the lowest. (Table 2; Figure 2). Supplementary File S3 shows the important methodology for guideline development of included CPGs.

**TABLE 2:** AGREE II Domain scores for included guidelines.

Guideline	Scope and Purpose (%)	Stakeholder Involvement (%)	Rigor of Development (%)	Clarity of Presentation (%)	Applicability (%)	Editorial Independence (%)	Mean Score (%)
Qiu, HB 2021 (Association SoCRDoCM, 2021)	72.22	33.33	52.08	83.33	4.17	50.00	49.19
Collins, T. 2020 (Collins et al., 2020)	83.33	50.00	43.75	77.78	20.83	33.33	51.50
Chou, C.C. 2018 (Chou et al., 2018)	66.67	44.44	37.50	66.67	16.67	33.33	44.21
Qu, JM 2018 (Infectious disease group RmboCMA, 2018)	83.33	38.89	20.83	72.22	16.67	0	38.66
Lenoe, M. 2018 (Leone et al., 2018)	72.22	50.00	37.50	77.78	12.50	50.00	52.78
Torres, A. 2017 (Torres et al., 2017)	83.33	77.78	79.17	94.44	41.67	50.00	71.06
Mikasa, K. 2016 (Mikasa et al., 2016)	72.22	44.44	35.42	94.44	25.00	50.00	53.59
Kalil, A.C. 2016 (Kalil et al., 2016)	83.33	77.78	68.75	94.44	50.00	83.33	74.54
Mehta, Y. 2014 (Mehta et al., 2014)	66.67	44.44	16.67	83.33	4.17	33.33	41.44
Klompas, M. 2014 (Klompas et al., 2014)	66.67	55.56	27.08	94.44	20.83	50.00	52.43
Alvarez-Lerma, F. 2014 (Álvarez Lerma et al., 2014)	61.11	27.78	50.00	94.44	20.83	83.33	56.25
Li, YM 2013 (Association CcmboCM, 2013)	61.11	44.44	43.75	94.44	4.17	0	41.32
Gupta, D. 2012 (Gupta et al., 2012)	61.11	38.89	62.50	94.44	50.00	83.33	65.05
Median score	72.22	44.44	43.75	94.44	20.83	50.00	52.43
Interquartile range (IQR)	(63.89,83.33)	(38.89,52.78)	(31.25,57.29)	(77.78,94.44)	(8.34,33.34)	(33.33,66.67)	(42.83,60.65)

#### 3.2.1 Scope and Purpose

The median score and interquartile range (IQR) of this domain was 72.22% (63.89%,83.33%), The highest score in this domain was 83.33% (Kalil et al., 2016; Torres et al., 2017; Infectious disease group RmboCMA 2018; Collins et al., 2020), and the lowest score was 61.11% (Gupta et al., 2012; Association CcmboCM 2013; Álvarez Lerma et al., 2014). The overall score in this field is high. All guidelines had clear overall objectives. The main problem is that the description of the target population is not clear, only four guidelines (Kalil et al., 2016; Torres et al., 2017; Infectious disease group RmboCMA 2018; Collins et al., 2020) specifically described the target population.

#### 3.2.2 Stakeholder Involvement

The median score and IQR of the stakeholder involvement domain was 44.44% (38.89%,52.78%), The highest score in this domain was 77.78% (Kalil et al., 2016; Torres et al., 2017), and the lowest score was 27.78% (Álvarez Lerma et al., 2014). Two guidelines (15.38%) scored over 60% (Kalil et al., 2016; Torres et al., 2017). Five guidelines included methodologists in evidence synthesis and guideline development (Association CcmboCM 2013; Klompas et al., 2014; Kalil et al., 2016; Torres et al., 2017; Collins et al., 2020). No guideline reported the involvement of patients or patient representatives, but the ERS 2017 guideline did provid a suggested interpretation of recommendations by the targeted stakeholders including patients, clinicians, and health policy makers (Torres et al., 2017).

#### 3.2.3 Rigor of Development

The median score and IQR of the rigor of development domain was 43.75% (31.25%,57.29%). The highest score in this domain was 79.17% (Torres et al., 2017), and the lowest score was 16.67% (Mehta et al., 2014). Only two guidelines (15.38%) scored over 60% (Gupta et al., 2012; Torres et al., 2017). The overall score in this field is low because of a lack of systematic methods for reporting the searching or evaluation of evidence.

#### 3.2.4 Clarity of the Presentation Domain

The median score and IQR of the clarity of the presentation domain was 94.44% (77.78%,94.44%). The highest score in this domain was 94.44% (Gupta et al., 2012; Association CcmboCM 2013; Klompas et al., 2014; Álvarez Lerma et al., 2014; Kalil et al., 2016; Mikasa et al., 2016; Torres et al., 2017), and the lowest score was 66.67% (Chou et al., 2018). All guidelines scored over 60%. The overall score in this field is the highest in six fields, and the quality of methodology is the best. All guidelines clearly describe each item in this field.

#### 3.2.5 Applicability Domain

The median score and IQR of the clarity of the applicability domain was 20.83% (8.33%,33.34%), which is the lowest score of all items. The highest score in this domain was 50.00% (Gupta et al., 2012; Kalil et al., 2016), and the lowest score was 4.17% (Association CcmboCM 2013; Mehta et al., 2014; Association SoCRDoCM 2021). No guideline scored over 60%. Most guidelines do not consider potential obstacles to implementation. The ERS 2017 guideline provided pocket guidelines in the supplementary files and added “implementation considerations” for every recommendation in the pocket guidelines (Torres et al., 2017).

#### 3.2.6 Editorial Independence Domain

The median score and IQR of the editorial independence domain was 50% (33.3%, 66.67%). The highest score in this domain was 83.33% (Gupta et al., 2012; Álvarez Lerma et al., 2014; Kalil et al., 2016), and the lowest score was 0% (Association CcmboCM 2013; Infectious disease group RmboCMA 2018). Three CPG (23.08%) scored over 60% (Gupta et al., 2012; Álvarez Lerma et al., 2014; Kalil et al., 2016). Six guidelines (Gupta et al., 2012; Mehta et al., 2014; Álvarez Lerma et al., 2014; Kalil et al., 2016; Chou et al., 2018; Collins et al., 2020) stated that the sponsor’s views had no impact on the recommendation, and five guidelines (Association CcmboCM 2013; Mehta et al., 2014; Chou et al., 2018; Infectious disease group RmboCMA 2018; Collins et al., 2020) did not mention the conflict of interest for the members of the formulation team.

### 3.3 Quality Assessment Comparison of Guidelines Developed With and Without GRADE System

All guidelines reported explicit grading for the strength of the recommendations. Nine guidelines reported that they used the Grading of Recommendations Assessment, Development and Evaluation (GRADE) tool for evaluating the quality of evidence and forming the final recommendations (Association CcmboCM 2013; Mehta et al., 2014; Álvarez Lerma et al., 2014; Kalil et al., 2016; Torres et al., 2017; Chou et al., 2018; Leone et al., 2018; Collins et al., 2020; Association SoCRDoCM 2021). Two guidelines (Mikasa et al., 2016; Infectious disease group RmboCMA, 2018)used a self-defined grading system, and one guideline (Klompas et al., 2014) used a combined grading system incorporating GRADE and the Canadian Task Force on Preventive Health Care and one guideline (34) use a modified GRADE system. (Table 3). We compared the quality of the guidelines developed with and without GRADE system. SPSS 23.0 software was used for statistical analysis, and *p* < 0.05 was considered a statistically significant difference. The results showed that there was no significant difference in the six domains of AGREE II. See Supplementary File S4 for details.

**TABLE 3:** Grading system of evidence and recommendation.

Guideline	Grading System Used	Description of Evidence	Description of Recommendation
Qiu, HB 2021 (Association SoCRDoCM, 2021)	GRADE	High; Moderate; Low; Very low	Strong; Weak
Collins, T. 2020 (Collins et al., 2020)	GRADE	High (1); Moderate (2); Low (3); Very low (4)	Strong; Moderate; Weak
Chou, C.C. 2018 (Chou et al., 2018)	GRADE	High [A]; Moderate [B]; Low [C]; Very low [D]	Strong [1]; Weak [2]
Qu, JM 2018 (Infectious disease group RmboCMA, 2018)	Self-defined	High(I); Moderate (II); Low (III)	Strong(A); Moderate(B); Weak(C)
Lenoe, M. 2018 (Leone et al., 2018)	GRADE	Strong; Moderate; Weak; Very weak	GRADE 1+; GRADE 1-; GRADE 2+; GRADE 2-
Torres, A. 2017 (Torres et al., 2017)	GRADE	High; Moderate; Low; Very low	Strong; Weak
Mikasa, K. 2016 (Mikasa et al., 2016)	Self-defined	I (Randomized comparative study); II (Non-randomized comparative study); III (Case report); IV (Specialist’s opinion)	A (strongly recommended); B (general recommendation), C (comprehensive evaluation by the attending physician)
Kalil, A.C. 2016 (Kalil et al., 2016)	GRADE	High; Moderate; Low; Very low	Strong; Weak
Mehta, Y. 2014 (Mehta et al., 2014)	GRADE	High (A) to very low (C)	Strong (grade 1); weak (grade 2)
Klompas, M. 2014 (Klompas et al., 2014)	GRADE and Canadian Task Force on Preventive Health Care	High(I); Moderate (II); Low (III)	Basic practices; Special approaches; Generally not Recommended; No recommendation
Alvarez-Lerma, F. 2014 (Álvarez Lerma et al., 2014)	GRADE	High; Moderate; Low; Very low	Strong; Weak
Li, YM 2013 (Association CcmboCM, 2013)	GRADE	High(A); moderate(B); low(C); very low(D)	Strong (1); Weak (2)
Gupta, D. 2012 (Gupta et al., 2012)	Modified GRADE system	Level 1; Level 2; Level 3; Useful practice point	GRADE A; GRADE B

GRADE: Grading of Recommendations Assessment, Development and Evaluation.

### 3.4 Recommendations on Drug Therapy for Prevention of VAP

We extracted 21 recommendations regarding drug therapy for prevention of VAP from 13 guidelines, including 5 strong recommendations, 5 moderate recommendations, 5 weak recommendation, 3 special approaches, 2 definitively not recommended, and one no formal recommendation. VAP has specific risk factors and pathogenesis. The recommendations that resulted from interpretation of the evidence varied among guidelines. Supplementary File S5 shows the recommendations on drug therapy for prevention, and Table 4 describes the chronological trend of recommendations on drug therapy for prevention of VAP.

**TABLE 4:** Chronological trend of recommendations on drug therapy for prevention of VAP.

Guidelines	Enteral Nutrition (SOR/LOE)	SOD (SOR/LOE)	SDD (SOR/LOR)	Chlorhexidine (SOR/LOE)	Probiotics (SOR/LOE)	Ulcer Prophylaxis (SOR/LOE)	Aerosol Inhalation (SOR/LOE)
Collins, T. 2020 (Collins et al., 2020)	——	——	——	Moderate/High	——	——	——
Qu, JM 2018 (Infectious disease group RmboCMA 2018)	Moderate/Moderate	Moderate/Moderate	Moderate/Moderate	Strong/Moderate	Moderate/Moderate	Moderate/Moderate	——
Torres, A. 2017 (Torres et al., 2017)	——	Weak/Low	——	No formal recommendation	——	——	——
Mehta, Y. 2014 (Mehta et al., 2014)	——	——	——	Strong/High	——	——	——
Klompas, M. 2014 (Klompas et al., 2014)	—/Moderate	—/High	——	—/Moderate	—/Moderate	—/Moderate	——
Alvarez-Lerma, F. 2014 (Álvarez Lerma et al., 2014)	——	Strong/High	Strong/High	Strong/Moderate	——	——	——
Li, YM 2013 (Association CcmboCM 2013)	Weak/Moderate	Weak/Moderate	Weak/Moderate	Strong/Low	Weak/Moderate	——	Weak/Low

Strongly recommended Moderate recommended Weakly recommended; Recommended (not have the SOR) Strongly not recommended Moderate not recommended Weakly not recommended; Not recommended (not have the SOR, or no formal recommendation).SDD: selective digestive decontamination; SOD: selective oral decontamination; SOR: Strength of recommendation; LOE: Level of evidence.

#### 3.4.1 Enteral Nutrition

There were 3 recommendations related to enteral nutrition for the prevention of VAP. Two Chinese guidelines (Association CcmboCM 2013; Infectious disease group RmboCMA 2018) recommended that early enteral nutrition is superior to parenteral nutrition as it can promote intestinal peristalsis, help to maintain the integrity of intestinal mucosal structure and barrier function, reduce pathogen colonization and bacterial translocation. The SHEA 2014 guideline (Klompas et al., 2014) did not recommend parenteral nutrition for VAP prevention, because it will reduce neither the incidence of VAP nor the duration of mechanical ventilation, hospital stay or mortality.

#### 3.4.2 Selective Oral Decontamination (SOD) or Selective Digestive Decontamination (SDD)

5 guidelines (Association CcmboCM 2013; Klompas et al., 2014; Álvarez Lerma et al., 2014; Torres et al., 2017; Infectious disease group RmboCMA 2018) made the suggestions for the use of SOD or SDD. The CMA 2013 guideline (Association CcmboCM 2013) and the SSICM 2014 guideline (Álvarez Lerma et al., 2014) recommend the use of SOD or SDD to prevent VAP, because it can decrease the rate of VAP mortality, although it had no effect on the time of mortality or length of mechanical ventilation. The SHEA 2014 guideline (Klompas et al., 2014) and the ERS 2017 guideline (Torres et al., 2017) advocated the use of SOD and avoidance of SDD, because most studies were conducted in countries or settings with low levels of antibiotic resistance, effectiveness of SOD or SDD in settings with high levels of antibiotic resistance has not been systematically assessed. Also, the potential effects of antibiotic use on antimicrobial resistant infections are inconclusive. The CMA 2018 guideline (1) did not provide explicit recommendations and only stated that SDD may increase the risk of drug-resistant bacterial infections, but there were no long-term follow-up studies; therefore, the Chinese guideline stressed the cautious use of SOD or SDD after weighing the advantages and disadvantages.

#### 3.4.3 Chlorhexidine

7 guidelines (Association CcmboCM 2013; Klompas et al., 2014; Mehta et al., 2014; Álvarez Lerma et al., 2014; Torres et al., 2017; Infectious disease group RmboCMA 2018; Collins et al., 2020) related to antiseptic oral rinse or the use of chlorhexidine. Four guidelines (Association CcmboCM 2013; Klompas et al., 2014; Mehta et al., 2014; Álvarez Lerma et al., 2014) published between 2013 and 2014 recommend the use of chlorhexidine for oral care, there is evidence that its use as a gargle may help to reduce the risk of VAP; The ERS 2017 guideline (Torres et al., 2017) decided not to issue a recommendation on the use of chlorhexidine until more safety data have become available, due to the unclear balance between a potential reduction in pneumonia rate and a potential increase in mortality. Also, the BACCN 2020 guideline (Collins et al., 2020) recommend that using an antiseptic oral rinse after brushing can help reduce the risk of VAP but may increase the mortality risk, further studies found that the use of chlorhexidine to prevent VAP was effective in cardiothoracic ICU, but it was unclear in the non-cardiothoracic ICU population, they advise caution with the routine use of chlorhexidine as part of an oral care program. It can be seen that in contrast to with other countries, the current China guidelines recommend the prophylactic use of chlorhexidine.

#### 3.4.4 Prophylactic Probiotics

3 guidelines discussed the use of prophylactic probiotics. Two Chinese guidelines (Association CcmboCM 2013; Infectious disease group RmboCMA 2018) do not recommended that probiotics be routinely given for prevention; the SHEA 2014 guideline (Klompas et al., 2014) recommended administering prophylactic probiotics.

#### 3.4.5 Stress Ulcer Prophylaxis

Two guidelines (Klompas et al., 2014; Infectious disease group RmboCMA 2018) have recommendations on stress ulcer prophylaxis, both the Chinese guideline (Infectious disease group RmboCMA 2018) and the SHEA 2014 guideline (Klompas et al., 2014) state they are definitively not recommended for VAP prevention: interventions with good-quality evidence suggesting that they neither lower VAP rates nor decrease duration of mechanical ventilation, length of stay, or mortality.

#### 3.4.6 Aerosol Inhalation of Antibiotics

The CMA 2013 guideline (Association CcmboCM 2013) recommended that Patients on mechanical ventilation should not routinely use aerosol inhalation of antibiotics to prevent VAP.

### 3.5 Recommendations on Drug Treatment for VAP

We extracted 51 recommendations regarding drug treatment for VAP from 8 guidelines (Gupta et al., 2012; Association CcmboCM 2013; Kalil et al., 2016; Mikasa et al., 2016; Torres et al., 2017; Infectious disease group RmboCMA 2018; Leone et al., 2018; Association SoCRDoCM 2021), including 28 strong recommendations, 22 weak recommendations, and one strongly not recommended. These recommendations included empirical antibiotics for VAP, etiological treatment, and the length of a course of antibiotic therapy. Supplementary File S6 shows the recommendations on drug treatment, and Table 5 describes the chronological trend of recommendations on drug treatment of VAP.

**TABLE 5:** Chronological trend of recommendations on drug treatment of VAP.

Guideline	Empiric Treatment Recommendation (SOR/LOE)	Aerosolized Antibiotics Recommendation (SOR/LOE)	Duration of Antibiotic Therapy (SOR/LOE)
Qiu, HB 2021 (Association SoCRDoCM 2021)	——	For VAP/HAP patients infected with multidrug-resistant gram-negative bacteria, systemic antibiotics combined with aerosol inhalation antibiotics can be considered to improve the cure rate of pneumonia and the clearance rate of respiratory bacteria (Weak/Low)	——
Qu, JM 2018 (Infectious disease group RmboCMA 2018)	For HAP/VAP patients with risk factors of MDR *Pseudomonas aeruginosa* and other MDR gram-negative bacilli infection or high risk of death, the use of two different types of antibiotics in combination is recommended; For patients with HAP/VAP who are not critical/have no risk factors for MDR infection, a single antibiotic can be used in empirical treatment (Strong/Low)	——	——
Lenoe, M. 2018 (Leone et al., 2018)	——	The administration of nebulized colimycin (sodium colistimethate) and/or aminoglycosides is suggested in documented HAP due multidrug-resistant Gram-negative bacilli documented pneumonia established as sensitive to colimycin and/or aminoglycoside, when no other antibiotics can be used (based on the results of susceptibility testing) *Data are only available for VAP (GRADE 2+)	The antibiotic treatment for HAP for longer than 7 days is not recommended, including for non-fermenting Gram-negative bacilli, apart from specific situations (immunosuppression, empyema, necrotizing or abscessed pneumonia) * Data are only available for VAP (GRADE 1-)
Torres, A. 2017 (Torres et al., 2017)	It is recommended that empiric treatment regimens be informed by the local distribution of pathogens associated with VAP and their antimicrobial susceptibilities. (See Supplementary File S6 for details)	——	Using a 7–8-days course of antibiotic therapy is suggested in patients with VAP without immunodeficiency, cystic fibrosis, empyema, lung abscess, cavitation, or necrotizing pneumonia and with a good clinical response to therapy (Weak recommendation, moderate quality of evidence)
Kalil, A.C. 2016 (Kalil et al., 2016)	It is recommended that empiric treatment regimens be informed by the local distribution of pathogens associated with VAP and their antimicrobial susceptibilities. (See Supplementary File S6 for details)	Both inhaled and systemic antibiotics, rather than systemic antibiotics alone are suggested for patients with VAP due to gram-negative bacilli that are susceptible to only aminoglycosides or polymyxins (colistin or polymyxin B) (Weak recommendation, very low-quality evidence)	For patients with VAP, a 7-days course of antimicrobial therapy rather than a longer duration is recommended (Strong recommendation, moderate-quality evidence)
Li, YM 2013 (Association CcmboCM 2013)	The initial empirical anti-infective treatment of VAP patients is usually single drug anti-infective treatment with appropriate antibacterial spectrum; If the pathogen is multi drug resistant, the combination treatment of antibiotics can be selected (1B)	For pulmonary infection caused by multidrug-resistant non fermenting bacteria, when the effect of systemic anti infection treatment is poor, combined aerosol inhalation of aminoglycosides or polymyxin and other drugs can be considered (1C)	VAP anti infection course is generally 7–10 days. If the patient has poor clinical response, multi drug resistant bacterial infection or immune function defect, the treatment time can be appropriately prolonged (1B)
Gupta, D. 2012 (Gupta et al., 2012)	There is no evidence to suggest that combination therapy is superior to monotherapy (1A)	Aerosolized antibiotics (colistin and tobramycin) may be a useful adjunct to intravenous antibiotics in the treatment of MDR pathogens where toxicity is a concern and should not be used as monotherapy but should be used concomitantly with intravenous antibiotics (2A)	In patients with VAP due to *Pseudomonas*, *Acinetobacter*, and MRSA, a longer duration (14 days) of antibiotic course is recommended; In other patients with VAP who are clinically improving, a 7-days course of antibiotics is recommended (1A)

HAP: Hospital-acquired Pneumonia; VAP: Ventilator-associated Pneumonia; MRSA: Methicillin-resistant *Staphylococcus aureus*; MDR: Multidrug resistance; SOR: Strength of recommendation; LOE: Level of evidence.

#### 3.5.1 Empirical Antibiotics

We extracted 21 recommendations regarding empiric therapy for VAP from 5 guidelines (Gupta et al., 2012; Association CcmboCM 2013; Kalil et al., 2016; Torres et al., 2017; Infectious disease group RmboCMA 2018). For empiric therapy, all guidelines recommended that the empirical treatment plan should be determined according to the local distribution of pathogens associated with VAP and their antimicrobial susceptibilities, drug resistance rates vary widely between countries, regions, and hospitals. Two Chinese guidelines (Association CcmboCM 2013; Infectious disease group RmboCMA 2018), the ERS 2017 guideline (Torres et al., 2017) and the IDSA 2016 guideline (Kalil et al., 2016) recommended that the empirical antibiotic treatment usually adopts appropriate antibacterial spectrum single drug treatment. Narrow-spectrum antibiotics (ertapenem, ceftriaxone, cefotaxime, moxifloxacin, or levofloxacin) were suggested for patients with low risk of multidrug resistance (MDR) infection and early-onset VAP (Torres et al., 2017). But the NCCP 2012 guideline (Gupta et al., 2012) recommend combination therapy due to the high prevalence rates of MDR pathogens in late-onset VAP aiming to maximize the chances of appropriateness of the initial regimen.

If the pathogen is considered empirically to be multidrug resistant bacteria, antibiotic combination treatment can be selected. The IDSA 2016 guideline (Kalil et al., 2016) gave indications for empiric dual gram-negative and MRSA therapy and recommended vancomycin or linezolid as empirical antibiotics for MRSA treatment. Combination therapy and antibiotics for MRSA treatment were suggested by the CMA 2018 guideline when a high risk of MDR exists, and empirical agents and antibiotics are listed in a table in the guidelines, but unfortunately, no recommendations on strength was supplied in the guideline text (Infectious disease group RmboCMA 2018).

#### 3.5.2 Etiological Treatment

In our study, about 15 recommendations relate to the etiological treatment of VAP. If the infecting pathogen is identified, the corresponding antimicrobial treatment plan (narrow-spectrum or broad-spectrum, single drug or combination) should be formulated with reference to the results of *in vitro* drug sensitivity tests. The IDSA 2016 guideline (Kalil et al., 2016), CMA 2018 guideline (Infectious disease group RmboCMA 2018) and IDST 2018 guideline (Chou et al., 2018) give detailed treatment plans for the etiological treatment of VAP, but the CMA 2018 guideline and the IDST 2018 guideline did not give any recommendation on strength.

*Acinetobacter* Baumann was found to be susceptible to sulbactam (SBT) and ampicillin (ABPC) which was recommended as a first-choice drug for respiratory infectious diseases (not VAP alone) in the JAID (Mikasa et al., 2016)guideline. The CMA 2018 guideline also recommended the use of SBT and ABPC, but for *Acinetobacter Baumann* infection (extensively-drug resistant or pan drug resistant), a combined regimen (sulbactam combined with polymyxin, tigecycline, or doxycycline) should be used. The IDSA 2016 guideline cautioned against the use of tigecycline in patients with hospital-acquired pneumonia (HAP) or VAP caused by *Acinetobacter* species, however they did not recommend any specific drug for *Acinetobacter* Baumann. This was based on evidence synthesis which indicated that the dose currently recommended on the label of tigecycline worsened clinical outcomes compared with several other therapies. The panel’s strong caution against tigecycline, despite low-quality evidence, was intended to emphasize the importance of avoiding potentially harmful therapies, particularly when alternative choices exist. This is in sharp contrast to the Chinese guidelines.

#### 3.5.3 Duration of Antibiotic Treatment

Five guidelines (Gupta et al., 2012; Association CcmboCM 2013; Kalil et al., 2016; Torres et al., 2017; Leone et al., 2018) covering 7 recommendations relate to the duration of antibiotic treatment. In the IDSA 2016 guideline (Kalil et al., 2016) and the SFAR 2018 guideline (Leone et al., 2018), a 7-days course of antimicrobial treatment rather than one of longer duration was strongly recommended. In the ERS 2017 guideline (Torres et al., 2017), a weak recommendation was given to using a 7–8-days course of antibiotic therapy. The CMA 2013 guideline (Association CcmboCM 2013) and the NCCP 2012 guideline (Gupta et al., 2012)points out that generally the normal length for a course of anti-infective drugs for VAP is 7–10 days. If the patient has poor clinical response, multidrug resistant bacterial infection or deficient immune function, the treatment time can be appropriately prolonged. We noted that none of the recommendations were based on evidence ranked as “strong quality”, even though they were both derived from evidence in many systematic reviews or meta-analyses. The IDSA 2016 guideline reported the advantages of a short-course regimen, which decreases antibiotic exposure and antibiotic resistance without increasing mortality or recurrent disease, and the decreased antibiotic exposure almost certainly reduces costs and side effects (Kalil et al., 2016). Longer courses may still be appropriate in some circumstances where the patient has a delayed clinical response. However, different patients can have treatment courses of variable length. Many guidelines suggest that we should consider the factors of both the host and the pathogen and make a comprehensive individualized judgment in combination with the clinical reaction and laboratory examination results.

#### 3.5.4 Aerosol Inhalation Antibiotic Therapy

Five guidelines (Gupta et al., 2012; Association CcmboCM 2013; Kalil et al., 2016; Leone et al., 2018; Association SoCRDoCM 2021) relate to the Aerosol inhalation antibiotic therapy. Both the Chinese guidelines (Association CcmboCM 2013; Association SoCRDoCM 2021) and other guidelines recommended that, for VAP patients infected with multidrug-resistant gram-negative bacteria, systemic antibiotics combined with aerosol inhalation antibiotics can be considered to improve the cure rate of pneumonia and the clearance rate of respiratory bacteria. It is necessary to make a comprehensive assessment of the dose, administration mode, adverse reactions and other factors associated with inhaled antibiotics in order to weigh the advantages and disadvantages.

## 4 Discussion

### 4.1 The Quality of Guidelines Needs to Be Improved in the Future

Since October 2016, the National Health Commission of the People’s Republic of China has initiated work to construct a national clinical practice guideline database aiming to promote CPG development, dissemination, and implementation in China. CPGs aim to formulate specific, explicit recommendations that, if properly adopted in clinical settings, will produce better outcomes for patients, and promote cost-effective practices (WHO 2018). The preponderance of CPGs developed by various organizations on similar or the same topic has been increasing throughout the world. All guidelines included in this study were evidence-based. The results show that the quality of the guidelines assessed was generally modest but varied between different organizations. The IDSA, SFAR and international ERS guidelines tended to have higher scores than others.

The methodologist plays a key role in guideline development meetings by helping the guideline development group formulate recommendations informed by the evidence in a transparent and explicit manner (WHO 2018). Over the past few years, we have seen a substantial increase in the number of guideline groups introducing and using the GRADE tool and in the number of options available within GRADE (Shekelle 2018). Usually, different guidelines used different scales or systems to evaluate or rate the quality and strength of evidence and recommendations (Harpole et al., 2003). In this study, although most of the included guidelines stated that they had used the GRADE system to assess and rate evidence quality and recommendation level, the assessors did not think that all guidelines used the GRADE system correctly. GRADE is based on the belief that recommendations should be based on a systematic review of the scientific literature guided by specific questions relating to the intervention, exposure, or approach under consideration (WHO 2018). GRADE evidence profiles should generally be part of the final report of the systematic review and contain an assessment of the evidence quality and a summary of findings across studies for each critical and important outcome and every key question (Holger Schünemann 2013). The guideline development group used evidence summaries as the basis for group discussions and to formulate recommendations.

The low score in the field of applicability indicates that the guideline expert group regards the development and implementation of the guideline as a separate issue and does not pay enough attention to the potential obstacles in its promotion and dissemination (Alonso-Coello et al., 2010). When reporting guidelines, the guideline team should provide tools such as charts applying recommendations to practice to facilitate implementation (WHO 2018).

Guideline recommendations should be based on the balance between the estimated costs of the interventions or services and their expected benefits compared with an alternative (Álvarez Lerma et al., 2014). Although formally assessing the cost effectiveness of an intervention, service or program can help decision-makers ensure that maximum gain is achieved from limited resources, economic evaluation evidence has rarely been cited in the guidelines we included. Unfortunately, drug recommendations for VAP were seldom based on systematic economic evaluation. Prices of different antimicrobial agents vary widely; cost-utility analysis is needed for a rational recommendation. Economic evaluation should start during guideline scoping and development of guideline questions especially those concerned with economic outcomes.

### 4.2 Recommendation Changes and Trends of Drug Therapy for Prevention

At present, VAP prevention measures focus on the pathogenesis to reduce the occurrence of VAP and improve the prognosis of patients. The recommendations for preventing VAP for these guidelines include non-drug prevention and drug prevention. Non-drug prevention mainly includes semi-recumbent position; use of new endotracheal intubation and subglottic secretion drainage; reducing the use of invasive ventilation, shortening the time of invasive ventilation, and limiting the use of narcotic drugs (Li Bassi et al., 2017; Infectious disease group RmboCMA 2018).

For drug prevention of VAP, we found that some disparities remain among the included guidelines. When there are substantial differences in major recommendations of guidelines, patients and clinical practitioners may question the validity which may then lead to poor adherence and implementation (Shekelle 2018). VAP drug prevention forms a special genre, it is challenging to explain the VAP prevention literature, because many measures have been reported to reduce the incidence rate of VAP, but the limitations of its diagnostic criteria make it difficult to identify the true effectiveness of preventative strategies.

At present, there are still many disputes about the specific scheme of selective purification and its clinical application in areas with different antibiotic resistance levels (Wittekamp et al., 2018; Hurley 2020; Rommes et al., 2020). Although several Randomized Controlled Trial (RCT) studies show that selective purification will not lead to the increase of antibiotic resistance rate, many ICU centers around the world are still cautious about its clinical application (de Smet et al., 2009; Oostdijk et al., 2014; Wittekamp et al., 2018). However, another SDD trial (clinicaltrials.gov NCT02389036) is being conducted in Canada, the UK and Australia (countries with moderate or above antibiotic resistance) which is conducting a concurrent cohort study alongside a randomized trial to assess the impact of SDD on antibiotic resistance patterns. Therefore, the results of this program will provide us with more information about the use of SDD in this population (Francis et al., 2014).

Recent studies have also questioned the efficacy and safety of oral chlorhexidine. Although there is evidence that its use as a gargle may help to reduce the risk of VAP, but this is unclear in the non-cardiothoracic ICU population (Houston et al., 2002; Segers et al., 2006; Labeau et al., 2011; Cuccio et al., 2012; Hua et al., 2020). Some studies have reported that oral care with chlorhexidine may increase mortality, possibly because some patients may inhale some preservatives that cause acute lung injury (Price et al., 2014; Klompas et al., 2016; Klompas 2017; Deschepper et al., 2018; Harris et al., 2018). A recent randomized trial show that in mechanically ventilated ICU patients, no benefit was observed for de-adoption of chlorhexidine and implementation of an oral care bundle on ICU mortality or time to discharge but may have improved oral health (Dale et al., 2021).

The application of probiotics in patients with mechanical ventilation is still controversial. Two recent meta-analysis have shown that the application of probiotics helped to prevent VAP without impacting the length of ICU stays or mortality (Su et al., 2020; Zhao et al., 2021). However, another recent meta-analysis has reached the opposite conclusion (Batra et al., 2020). By analyzing the above research, we found that the main problem is the difference of inclusion criteria. Probiotics may be an attractive intervention in the prevention of ventilator-associated pneumonia in adult hospitalized patients. However, the certainty of the evidence on its cost-effectiveness is very low. Future randomized controlled trials of probiotics should include cost data to inform bedside practice, clinical guidelines, and medical policies (Lau et al., 2020).

### 4.3 Recommendation Changes and Trends of Drug Therapy for Treatment

The treatment of VAP includes two aspects: first, empirical treatment, which needs to consider the severity of the patient’s disease, suspected pathogens and MDR risk factors; second, etiological treatment. Indeed, some evidence used by those guidelines was derived from the same trials or systematic reviews, meaning that they are based on shared evidence. Variations could be the result of differences in data interpretation or the indication of available resources, while actual recommendations could still be tailored to local and cultural contexts. Nonetheless, one needs to be cautious when considering guidelines for local use and should make sure that the clinical data and evidence are in harmony with the clinical judgment.

The pathogens found in VAP patients are mainly Gram-negative bacteria (Abd-Elmonsef et al., 2018). Antibiotics active against Gram-negative bacteria should be preferred in the early stage of infections with unknown bacterial. Currently, we need to avoid the excessive use of antibiotics as their inappropriate application will increase the mortality. When treating VAP, it is also necessary to avoid the application of broad-spectrum antibiotics as this will induce drug resistance. Antimicrobial flora and resistance patterns can vary considerably between and within countries, regions, hospitals, ICUs in a hospital, and specimen sources. In China, It is reported that the isolation rate of *acinetobacter* baumannii in the VAP pathogen spectrum was as high as 35.7–50.0%, followed by *Pseudomonas aeruginosa* and *Staphylococcus aureus*, and the isolation rate of *Acinetobacter* Baumann has been reportedly increasing year by year (Infectious disease group RmboCMA 2018). By the 2014–2019 Chinese Antimicrobial Resistance Surveillance of Nosocomial Infection, the detection rates of MRSA, Methicillin Resistant Coagulase-Negative Staphylococci (MRCNs) and vancomycin resistant enterococci had decreased, but the drug resistance of *Acinetobacter* Baumann to various antibiotics was at a serious level (Network Nbrm 2021). However, surveillance studies in the United States suggested that the organisms most associated with VAP have been *S. aureus* (approximately 20–30% of isolates), *P. aeruginosa* (approximately 10–20% of isolates), enteric gram-negative bacilli (approximately 20–40% of isolates), and *Acinetobacter* Bahmani (approximately 5–10% of isolates) (Sievert et al., 2013). Understanding the distribution and drug resistance of local pathogens in VAP patients is helpful to formulate a scientific empirical treatment plan, which is very important in reducing the mortality of VAP patients and delaying the occurrence of bacterial drug resistance.

Antimicrobial stewardship is an important issue; however, the approach to manage it differs considerably in different countries. Currently more and more attention has been paid to the deterioration of VAP-associated death rates in China. The use of antibiotics in China faces severe challenges, and in recent years the National Health Commission of the People’s Republic of China has continued to issue notices on further strengthening the management of the clinical application of antimicrobial drugs to curb bacterial resistance (Commission GOotnhaFP 2017; Commission GOoNHaH GOoNHaH 2020). The aim is to improve the level of diagnosis and treatment of infectious diseases, improve testing, promote the accurate use of antibacterial drugs, and rely on information construction to help the scientific management of antibacterial drugs. Through the use of these different measures, the level of scientific management of antibacterial drugs will be continuously improved.

VAP will continue to be the main infection in ICU in the next decade (Xie et al., 2019). This paper evaluates the quality of VAP guidelines and summarizes and analyzes the drugs recommended for prevention and treatment. The differences in drugs for prevention and treatment of VAP in different countries have been compared. At the same time, according to the publication time of different guidelines, the changing trend of VAP prevention and treatment across the years has been summarized. We hope that the present study contributes to the prevention and control of VAP. Therefore, for the prevention of VAP, appropriate antibiotics should be selected according to the characteristics of drug resistance in different countries. In addition, due to the increase of antibiotic resistance, the development of new drugs against MDR pathogens is also very urgent.

### 4.4 Strengths and Limitations

Our study possesses several strengths. First, our research team consisted of methodologists with full experience in the development and assessment of CPGs and clinical experts and obtained consensus from all appraisers to ensure the reliability of our conclusions. Second, we conducted a thorough systematic literature search. Third, we extracted and compared recommendations for drug use for VAP.

Nevertheless, several limitations should be noted. First, we only assessed guidelines published in the English and Chinese languages and on some important professional society websites, which may not represent all the guidelines for VAP. Guidelines published in other ways (i.e., books, booklets, other websites, or health institution documents) may have been omitted, which may have introduced bias into our assessment. Second, we attached relatively more weight to the quality of guideline development than to whether the recommendations were feasible in our specific practice environments or matched a particular clinical practice We extracted recommendations on drug therapy for VAP prevention and treatment; however, many guidelines were developed for both HAP and VAP, and their recommendations did not differentiate between the two mostly because some guidelines considered VAP to be a special type of HAP, and primary studies included a mix of HAP and VAP samples. In this study, we recognized that patients with HAP and VAP belong to 2 distinct groups and extracted the recommendations that only applied to VAP or recommendations that were described as pertinent to VAP/HAP with evidence indicating the recommendation came from VAP-related research.

## 5 Conclusion

In conclusion, there is considerable variability in the development process and reporting of VAP guidelines, although the principles for guideline development have been described. The experience of the organization and experts in assessing evidence and developing guidelines may explain higher scores for some items. There were substantial differences in some recommendations of VAP guidelines. For the prevention and treatment of VAP, local microbial epidemiology and antibiotic sensitivity must be considered, along with economic issues. The most effective clinical practice guidelines should incorporate the current best evidence and place these in the context of local patterns of drug resistance. Recommendations should be regularly revised as new evidence emerges.

## Data Availability

The original contributions presented in the study are included in the article/Supplementary Material, further inquiries can be directed to the corresponding authors.
