# Supplementary material for: Management of Ventilator-Associated Pneumonia: Quality Assessment of Clinical Practice Guidelines and Variations in Recommendations on Drug Therapy for Prevention and Treatment
Source: Front Pharmacol. 2022 May 20;13:903378. doi: 10.3389/fphar.2022.903378 (PMC9163435; doi:10.3389/fphar.2022.903378)
Supplement: Supplementary file 1 [file Table1.DOCX]

**#1** "Pneumonia, Ventilator-Associated"[MeSH Terms] OR "Ventilator-Associated Pneumonia"[Title/Abstract]

**#2** "Respiratory Tract Infections"[MeSH Terms] OR "Respiratory Tract Infections"[Title/Abstract] OR "Infections, Respiratory"[Title/Abstract] OR "Upper Respiratory Tract Infections"[Title/Abstract] OR "Upper Respiratory Infections"[Title/Abstract] OR "Respiratory Tract Infection"[Title/Abstract] OR "Upper Respiratory Tract Infection"[Title/Abstract] OR "Upper Respiratory Infection"[Title/Abstract]

**#3** Pneumonia [MeSH Terms] OR Pneumonia*[Title/Abstract] OR "Lobar Pneumonias"[Title/Abstract] OR "Experimental Lung Inflammations"[Title/Abstract] OR Pneumonitis [Title/Abstract] OR Pneumonitides[Title/Abstract] OR "Pulmonary Inflammation"[Title/Abstract] OR "Lung Inflammations"[Title/Abstract] OR "Lobar Pneumonia"[Title/Abstract] OR "Experimental Lung Inflammation"[Title/Abstract] OR "Pulmonary Inflammation"[Title/Abstract] OR "Lung Inflammation"[Title/Abstract]

**#4** "Respiration, Artificial"[MeSH Terms] OR "Respiration, Artificial"[Title/Abstract] OR "Artificial Respirations"[Title/Abstract] OR "Mechanical Ventilations"[Title/Abstract] OR "artificial airways"[Title/Abstract] OR "Artificial Respiration"[Title/Abstract] OR "Mechanical Ventilation"[Title/Abstract] OR "artificial airway"[Title/Abstract]

**#5: #2 AND #3 AND #4**

**#6: #1 OR #5**

**#7** Guideline [Publication Type] OR Practice Guideline [Publication Type] OR "Guidelines as Topic"[MeSH Terms] OR "Guidelines as Topic"[Title/Abstract] OR guideline*[Title/Abstract] OR guidance*[Title/Abstract] OR recommendation*[Title/Abstract] OR CPG*[Title/Abstract]

**#8: #6 AND #7**
