# Supplementary material for: Management of Ventilator-Associated Pneumonia: Quality Assessment of Clinical Practice Guidelines and Variations in Recommendations on Drug Therapy for Prevention and Treatment
Source: Front Pharmacol. 2022 May 20;13:903378. doi: 10.3389/fphar.2022.903378 (PMC9163435; doi:10.3389/fphar.2022.903378)
Supplement: Supplementary file 2 [file Table2.DOCX]

**Additional file 2 Search resources of professional society websites**

| **Title** | **Websites** |
| --- | --- |
| World Federation of Societies of Intensive and Critical Care Medicine, WFSICCM | http://www.world-critical-care.org/ |
| Canadian Critical Care Society, CCCS | http://www.canadiancriticalcare.org/ |
| Society of Critical Care Medicine, SCCM | http://www.sccm.org/ |
| European Society of Intensive Medicine, ESICM | https://www.esicm.org/ |
| Malaysian Society of Intensive Care, MSIC | http://www.msic.org.my/ |
| Australian and New Zealand Intensive Care Society, ANZICS | <http://www.anzics.com.au/www.anzics.com.au/index.html> |
| Intensive Care Society, ICS | http://www.ics.ac.uk/ |
| Chinese Society of Critical Care Medicine, CSCCM | http://www.csccm.org/cn/ |
| Intensive Care Society of Ireland, ICSI | http://www.intensivecare.ie/ |
| Scottish Intensive Care Society | https://www.scottishintensivecare.org.uk/ |
| Society of Intensive Care Medicine, SICM | https://sicm.org.sg/ |
| Emirates Intensive Care Society, EICS | http://www.eics.ae/ |
| Critical Care Society of South Africa, CCSSA | http://www.criticalcare.org.za/ |
| Saudi Critical Care Society, SCCS | http://www.sccs-sa.org/ |
| Indian Society of Critical Care Medicine, ISCCM | http://www.isccmdelhi.com/ |
| Belgian Society of Critical Care Medicine, SIZ | http://www.siz.be/ |
| American Association of Critical-Care Nurses, AACN | https://www.aacnnursing.org/ |
| American Thoracic Society, ATS | http://www.thoracic.org/ |
| British Thoracic Society, BTS | https://www.brit-thoracic.org.uk/ |
| European Respiratory Society, ERS | https://www.ersnet.org/ |
| American College of Chest Physicians, CHEST | https://www.chestnet.org/ |
| Infectious Diseases Society of America, IDSA | https://www.idsociety.org/ |
| European Society of Clinical Microbiology and Infectious Diseases, ESCMID | https://www.escmid.org/ |
| International Society for Infectious Diseases, ISID | https://www.isid.org/ |
| Australasian Society for Infectious Diseases, ASID | https://www.asid.net.au/ |
