# Supplementary material for: Management of Ventilator-Associated Pneumonia: Quality Assessment of Clinical Practice Guidelines and Variations in Recommendations on Drug Therapy for Prevention and Treatment
Source: Front Pharmacol. 2022 May 20;13:903378. doi: 10.3389/fphar.2022.903378 (PMC9163435; doi:10.3389/fphar.2022.903378)
Supplement: Supplementary file 3 [file Table5.DOCX]

**Additional file 5 Recommendations of drugs for prevention of VAP**

| **Guideline** | **Recommendations** | **Grade of recommendation** | **Evidence level** | **Evidence base** |
| --- | --- | --- | --- | --- |
| Qiu, HB 2021 | —— | —— | —— | —— |
| Collins, T. 2020 | Due to meta-analysis evidence, caution is advised at present with the routine use of chlorhexidine as part of an oral care program. | Moderate | High quality evidence | SRs /meta-analyses of RCTs; RCT; Consensus guideline. |
| Chou, C.C. 2018 | —— | —— | —— | —— |
| Qu, JM 2018 | enteral nutrition should be given for patients on mechanical ventilation. | Moderate | Moderate-quality evidence | Strategies; RCTs; meta-analysis of RCTs. |
|  | Routine oral health care（chlorhexidine or Povidone iodine，normal saline）is recommended for patients on mechanical ventilation. | Strong | Moderate-quality evidence | Clinical practice guideline. |
|  | SOD or SDD should be used cautiously after weighing the advantages and disadvantages. | Moderate | Moderate-quality evidence | Meta-analyses of RCTs; RCT; Clinical practice guidelines. |
|  | It is not recommended that probiotics be routinely given to prevent VAP. | Moderate | Moderate-quality evidence | Meta-analysis of RCTs; Clinical practice guidelines. |
|  | At present, it is considered that the use of acid inhibitors to prevent stress ulcer may increase the colonization of bacteria in gastrointestinal tract and airway, but it has no effect on the mortality of patients with VAP. Attention should be paid to the indications for its application. | Moderate | Moderate-quality evidence | Meta-analyses of RCTs. |
| Leone, M. 2018 | —— | **——** | **——** | —— |
| Torres, A. 2017 | The guideline panel decided not to issue a recommendation on the use of chlorhexidine to perform selective oral decontamination (SOD) in patients requiring mechanical ventilation until more safety data become available, due to the unclear balance between a potential reduction in pneumonia rate and a potential increase in mortality. | No formal recommendation | **——** | SRs/meta-analyses of RCTs; Network meta-analyses; RCTs. |
|  | the use of SOD, but not selective digestive decontamination (SDD) is suggested in settings with low rates of antibiotic-resistant bacteria and low antibiotic consumption (where low antibiotic consumption in the ICU is <1000 daily doses per 1000 admission days). | Weak | Low quality of evidence | SRs/meta-analyses of RCTs; SRs/meta-analyses of observational studies; Network meta-analyses; RCTs; Cross-sectional studies; Prospective clinical trials; Follow-up studies. |
| Mikasa, K. 2016 | —— | —— | —— | —— |
| Kalil, A.C. 2016 | —— | —— | —— | —— |
| Mehta, Y. 2014 | Strategies to reduce VAP: Daily oral care with chlorhexidine solution of strength 0.12%. | Strong | High quality evidence | Reviews |
| Klompas, M. 2014 | Interventions that decrease duration of mechanical ventilation, length of stay, and/or mortality but for which insufficient data on possible risks are available: Use selective decontamination of the oropharynx to decrease the microbial burden of the aerodigestive tract. | Special approaches | High | RCTs; Crossover studies; SRs/meta-analyses of RCTs. |
|  | Interventions that may lower VAP rates but for which there are insufficient data at present to determine their impact on duration of mechanical ventilation, length of stay, and mortality: Perform oral care with chlorhexidine. | Special approaches | Moderate-quality evidence | SRs/meta-analyses of RCTs. |
|  | Interventions that may lower VAP rates but for which there are insufficient data at present to determine their impact on duration of mechanical ventilation, length of stay, and mortality: Administer prophylactic probiotics. | Special approaches | Moderate-quality evidence | SRs/meta-analyses of RCTs. |
|  | Stress ulcer prophylaxis is definitively not recommended for VAP prevention: interventions with good-quality evidence suggesting that they neither lower VAP rates nor decrease duration of mechanical ventilation, length of stay, or mortality. | Definitively not recommend | Moderate-quality evidence | SRs/meta-analyses of RCTs. |
|  | Early parenteral nutrition is definitively not recommended for VAP prevention: interventions with good-quality evidence suggesting that they neither lower VAP rates nor decrease duration of mechanical ventilation, length of stay, or mortality. | Definitively not recommenced | Moderate-quality evidence | RCT |
| Alvarez-Lerma, F. 2014 | For the prevention of VAP, oral hygiene with chlorhexidine is recommended. | Strong | Moderate quality of evidence | SRs/meta-analyses of RCTs; Cohort studies; Cross-sectional studies. |
|  | For the prevention of VAP, SDD or SOD is recommended. | Strong | High quality of evidence | SRs/meta-analyses of RCTs; RCTs; Crossover studies; Prospective randomized animal studies. |
| Li, YM 2013 | It is suggested that selection of nasal tube feeding for nutritional support in patients on mechanical ventilation can reduce the incidence rate of VAP. | Weak | Moderate | RCTs; prospective cohort studies. |
|  | Oral care with chlorhexidine in patients on mechanical ventilation can reduce the incidence of VAP. | Strong | Low | Meta-analyses of RCTs; RCTs. |
|  | Nebulized inhaled antimicrobials are not routinely used to prevent VAP in mechanically ventilated patients. | Weak | Low | RCTs |
|  | Using SDD or SOD strategies to prevent VAP for patients with mechanical ventilation may be considered. | Weak | Moderate | RCTs |
|  | Routine application of intestinal probiotics to prevent VAP is not recommended for patients on mechanical ventilation. | Weak | Moderate | Meta-analyses of RCTs; RCTs; |
| Gupta, D. 2012 | —— | —— | —— | —— |

VAP: Ventilator-associated pneumonia; SDD: Selective Decontamination of the Digestive Tract; SOD: Selective Decontamination of the Oropharynx; SRs: Systematic Reviews; RCT: Randomized Controlled Trail.
