# Supplementary material for: Management of Ventilator-Associated Pneumonia: Quality Assessment of Clinical Practice Guidelines and Variations in Recommendations on Drug Therapy for Prevention and Treatment
Source: Front Pharmacol. 2022 May 20;13:903378. doi: 10.3389/fphar.2022.903378 (PMC9163435; doi:10.3389/fphar.2022.903378)
Supplement: Supplementary file 4 [file Table3.DOCX]

**Additional file 3 Important methodology for guideline development of CPGs included**

| **Guideline** | **Multidisciplinary cooperation** | **Sources of evidence** | **Criteria for selecting the evidence** | **The methods reaching agreement** | **Funding and disclosure of conflict of interest** |
| --- | --- | --- | --- | --- | --- |
| Qiu, HB 2021 | Specialists from department of intensive care unit. | PubMed, Embase, the Cochrane Library, Web of Science and three major Chinese academic databases. | RCT was preferentially included, and non-randomized controlled studies and simulation studies were included in the absence of RCT. | GRADE methodology | All members of the working group of this guideline have filled in the interest statement form, and there is no direct economic conflict of interest with this guide. |
| Collins, T. 2020 | An elected national board member for the BACCN who has experience in generating national guidelines and consensus documents. A consensus committee of critical care nurse experts from around the United Kingdom attend the meeting. | Relevant evidence was identified by searching PubMed (including Medical Subject Headings), the Cumulative Index of Nursing and Allied Health Literature (CINAHL) (including CINAHL Subject Headings), and Web of Science databases. | Primary and secondary research articles published in English prior to December 2018, were selected predominantly based on their relevance to oral care practices in adult critical care settings. | Each committee member had the opportunity to present their individual evaluations which were then discussed and clarified until a consensus was reached. A final consensus statement was agreed upon by all committee members after several rounds of comments and discussion which took place over 12 months. | A research grant was kindly provided by Stryker in supporting the publication of this document. |
| Chou, C.C. 2018 | A writing group, named the GREAT working group, was appointed in 2017 by the Infectious Diseases Society of Taiwan, with representative members from 13 medical centers in Taiwan. | —— | —— | Each author was assigned to review the literature for a single topic, evaluate the evidence, and determine the strength of the recommendations according to GRADE methodology. All panel members discussed given topics and recommendations during 5 face-to-face meetings to deliberate the statements and reach a consensus. An outside external panel of experts then reviewed the final guidelines at a consensus meeting. | This work was supported by Infectious Diseases Society of Taiwan (IDST), Taiwan, the Taiwan Society of Pulmonary and Critical Care Medicine (TSPCCM), Taiwan. Funding was also provided from the Medical Foundation in Memory of Dr. Deh-Lin Cheng, Taiwan. |
| Qu, JM 2018 | Pharmacologist, Infectious disease specialist, Critical care medicine specialist, foreign experts from America, Japan. | _____ | _____ | After repeated discussions among all members of the study group, a unified opinion was formed, and the opinions of experts in relevant fields at home and abroad were widely solicited. After many modifications, the final draft was finally finalized. | _____ |
| Lenoe, M. 2018 | Sixteen French speaking experts were selected by an organizing committee appointed by the guideline committees of each participating society. Two independent bibliographic experts analyzed the literature. | PubMed and Cochrane databases. | Over the last 10 years; written in English or in French. | Each recommendation was then evaluated by each expert rated using a scale from 1 (complete disagreement) to 9 (complete agreement). Collective rating was established according to a GRADE grid methodology. For a recommendation to be strong, at least 70% of participants had to have concordant opinions. Without strong agreement, recommendations were rephrased and resubmitted to reach a consensus. Consensus if required, by voting. | Each expert was required to file a conflict-of-interest disclosure prior to participation in establishing the guidelines. |
| Torres, A. 2017 | Specialists in respiratory medicine with expertise in the management of patients with lung infections, intensive care specialists, microbiologists, methodologists with experience in evidence synthesis and guideline development. | PubMed platform was used to search MEDLINE, The CENTRAL, the Cochrane Database of Systematic Reviews and the National Health Service’s Economic Evaluation Database were also searched to find additional studies and economic evaluations. | Human studies (systematic reviews, randomized clinical trials or observational studies) written in English. | Recommendations and their strength were decided by consensus and, if required, by voting. | D. Rigau acts as a methodologist for the European Respiratory Society. All other disclosures can be found alongside this article at erj.ersjournals.com. |
| Mikasa, K. 2016 | Specialists in the department of infectious diseases, department of pediatrics, department of respiratory medicine, department of pathogen, infection and immunity, department of laboratory medicine. | _____ | _____ | _____ | Detailed collecting information reporting about existed conflict of interest of developers without assessing and managing information. |
| Kalil, A.C. 2016 | Two chairs, 18 subject-matter experts which included specialists in infectious diseases, pulmonary medicine, critical care medicine, laboratory medicine, microbiology, and pharmacology as well as a guideline methodologist. | Electronic searches, panelists contacted experts and hand-searched journals, conference proceedings, reference lists, and regulatory agency websites. | Studies performed in adults and those published in English or containing an English abstract. No publication year limits were used. | Evidence summaries for each question were prepared by the panel members using the GRADE approach for rating the condense in the evidence. The summaries of evidence were discussed and reviewed by all committee members and edited as appropriate. | The IDSA and the ATS provided meeting facilities for face-to-face meetings, financial support for conference calls, and administrative support. Industry funding to support guideline development was not permitted; Detailed information about disclosure and management of potential conflicts of interest were reported. |
| Mehta, Y. 2014 | Authors come from Department of anesthesia critical care and pain. | _____ | _____ | _____ | Source of Support: Nil, Conflict of Interest: None declared. |
| Klompas, M. 2014 | Two experts in the prevention of HAIs to bisection panel leads. Expert panel members with broad healthcare epidemiology and infection prevention expertise were convened to review draft manuscripts and to provide input to each section panel. | _____ | _____ | _____ | Potential conflicts of interest. M.K. reports having received honoraria for lectures from Premier Healthcare Alliance. All authors report no conflicts of interest. |
| Alvarez-Lerma, F. 2014 | _____ | Published clinical trials, guidelines, systematic reviews, and meta-analyses. | _____ | Quantitative assessment by the 11 members of the panel considering the quality of the evidence, its safety, and its feasibility in Spanish ICUs. Finally, feasibility and cost criteria were applied, as recommended. | The Spanish Ministry of Health provided financial support for the meetings of the task force; Conflicts of interest: The authors declared that they have no competing interests. |
| Li, YM 2013 | Specialists in respiratory medicine； intensive care specialists; methodologists with experience in evidence synthesis and guideline development. | PubMed/Medline, Embase, The Cochrane Central Register of Controlled Trials (CENTRAL); CNKI, Wan fang Data | —— | The working group convened an expert seminar to discuss, vote and revise, and finally formulated the revised draft of the guide. When there were different opinions, it was resolved by voting. | —— |
| Gupta, D. 2012 | Departments of Internal Medicine, Microbiology, Pharmacology, and Radiodiagnosis. | PubMed, Embase; Website of Infectious IDSA, ATS, BTS, ERS. | —— | Discussions for grading of evidence and recommendations were held in four different groups and thereafter together in the joint meeting of all the groups. Final decisions in the joint group were based on a consensus approach on the majority voting. | Source of Support: Jointly sponsored by the Indian Chest Society and the National College of Chest Physicians (India), Conflict of Interest: None declared. |

GRADE: Grading of Recommendations Assessment, Development and Evaluation; RCT: Randomized Controlled Trial; HAIs: Hospital acquired infections; IDSA: Infectious Disease Society of America; ATS: American Thoracic Society; BTS: British Thoracic Society; ERS: European Respiratory Society; CENTRAL: The Cochrane Central Register of Controlled Trials.
