# Supplementary material for: Management of Ventilator-Associated Pneumonia: Quality Assessment of Clinical Practice Guidelines and Variations in Recommendations on Drug Therapy for Prevention and Treatment
Source: Front Pharmacol. 2022 May 20;13:903378. doi: 10.3389/fphar.2022.903378 (PMC9163435; doi:10.3389/fphar.2022.903378)
Supplement: Supplementary file 5 [file Table4.DOCX]

**Additional file 4 Quality assessment comparison guidelines developed with and without GRADE system**

|  | **GRADE system（mean±SD）** | **non-GRADE system（mean±SD）** | | **P-value** |
| --- | --- | --- | --- | --- |
| **Scope and Purpose (%)** | 72.22±9.21 | | 70.83±9.49 | 0.808 |
| **Stakeholder Involvement (%)** | 50.00±17.35 | | 44.45±7.86 | 0.559 |
| **Rigour of Development (%)** | 47.69±18.23 | | 36.46±18.36 | 0.328 |
| **Clarity of Presentation (%)** | 85.18±10.01 | | 88.89±11.11 | 0.563 |
| **Applicability (%)** | 19.45±16.54 | | 28.13±14.97 | 0.39 |
| **Editorial Independence (%)** | 46.29±26.06 | | 45.83±34.36 | 0.979 |
