# Supplementary material for: Management of Ventilator-Associated Pneumonia: Quality Assessment of Clinical Practice Guidelines and Variations in Recommendations on Drug Therapy for Prevention and Treatment
Source: Front Pharmacol. 2022 May 20;13:903378. doi: 10.3389/fphar.2022.903378 (PMC9163435; doi:10.3389/fphar.2022.903378)
Supplement: Supplementary file 6 [file Table6.DOCX]

**Additional file 6 Recommendations of drugs for treatment of VAP**

| **Guideline** | **Recommendations** | **Grade of recommendation** | **Evidence level** | **Evidence base** |
| --- | --- | --- | --- | --- |
| Qiu, HB 2021 | For VAP/HAP patients infected with multidrug-resistant gram-negative bacteria, systemic antibiotics combined with aerosol inhalation antibiotics can be considered to improve the cure rate of pneumonia and the clearance rate of respiratory bacteria. | Weak | Low | Meta-analyses of RCTs; RCT. |
| Collins, T. 2020 | —— | —— | —— | —— |
| Chou, C.C. 2018 | —— | —— | —— | —— |
| Qu, JM 2018 | Patients with HAP/VAP should receive empirical treatment with antibiotics as soon as possible. | Strong | Low-quality evidence | Observational studies. |
|  | The empirical treatment plan should be formulated according to the HAP/VAP pathogen spectrum and drug sensitivity test results of the hospital. | Strong | Low-quality evidence | Case-control study; Observational studies; clinical practice guidelines. |
|  | For HAP/VAP patients with high risk of MDR Pseudomonas aeruginosa and other MDR gram-negative bacilli infection or high risk of death, the use of two different types of antibiotics in combination is recommended; For patients with HAP/VAP who are not critical / have no risk factors for MDR infection, one antibiotic can be used in empirical treatment. | Strong | Low-quality evidence | —— |
|  | The treatment course for VAP should be changed to target therapy or step-down therapy as soon as possible (from combination therapy to single drug therapy, or from broad-spectrum antibiotics to narrow-spectrum antibiotics), where clear etiological results are obtained. | Weak | Low-quality evidence | RCTs; Observational studies; |
|  | Combined therapy with inhaled antibiotics and systemic antibacterial treatment can be tried where the following conditions are met: (1) HAP/VAP is caused by MDR Klebsiella pneumoniae, Pseudomonas aeruginosa, Acinetobacter Baumann, etc.; (2) The distribution of drugs in pneumonia was insufficient and the curative effect was poor; (3) The pathogens are sensitive to the selected antibiotics to be inhaled. | Weak | High-quality evidence | Clinical practice guidelines. |
| Lenoe, M. 2018 | Prolonging the antibiotic treatment for HAP, including for non-fermenting Gram-negative bacilli, apart from specific situations (immunosuppression, empyema, necrotizing or abscessed pneumonia) for more than 7 days is not recommended * Data are only available for VAP. | Strong recommendation against | High-quality evidence | Meta-analyses of RCTs. |
|  | Administering nebulized colimycin (sodium colistimethate) and/or aminoglycosides is suggested for documented HAP due multidrug-resistant Gram-negative bacilli documented pneumonia established as sensitive to colimycin and/or aminoglycoside, when no other antibiotics can be used (based on the results of susceptibility testing) *Data are only available for VAP. | Weak | Low-quality evidence | RCTs; Meta-analyses of RCTs; Meta-analyses of observational studies; Observational studies. |
| Torres, A. 2017 | Use of narrow-spectrum antibiotics (ertapenem, ceftriaxone, cefotaxime, moxifloxacin, or levofloxacin) in patients with suspected low risk of resistance and early-onset HAP/VAP is suggested. | Weak | Very low quality of evidence. | Prospective observational cohort study; prospective cohort study; observational retrospective or cohort studies. |
|  | Broad-spectrum empiric antibiotic therapy is recommended to target Pseudomonas aeruginosa and extended-spectrum à-lactamase- producing organisms, and, in settings with a high prevalence of Acinetobacter spp., in patients with suspected early-onset HAP/VAP who are in septic shock, in patients who are in hospitals with a high background rate of resistant pathogens present in local microbiological data and in patients with other (neoclassic) risk factors for MDR pathogens. | Strong recommendation | Low quality of evidence | Prospective observational cohort study; prospective cohort study; observational retrospective or cohort studies. |
|  | Initial empiric combination therapy for high-risk HAP/ VAP patients to cover Gram-negative bacteria is recommended including antibiotic coverage for MRSA in those patients at risk. | Strong recommendation | Moderate quality of evidence | Systematic review; RCTs observational studies; meta-analysis of randomized and observational studies. |
|  | If initial combination therapy is started, continuing with a single agent based on culture results is suggested and maintaining definitive combination treatment based on sensitivities should only be considered in patients with extensively drug-resistant (XDR, i.e., susceptible to only one or two classes of antibiotics)/pan-drug-resistant (PDR, i.e., not susceptible to any antibiotics) nonfermenting Gram-negative bacteria and carbapenem-resistant Enterobacteriaceae (CRE) isolates. | Weak recommendation | Low quality of evidence | systematic review; RCTs; observational studies; meta-analysis of randomized and observational studies. |
|  | A 7–8-day course of antibiotic therapy is suggested in patients with VAP without immunodeficiency, cystic fibrosis, empyema, lung abscess, cavitation, or necrotizing pneumonia and with a good clinical response to therapy. | Weak recommendation | Moderate quality of evidence | SRs /meta-analyses of RCTs; Randomized trials; Pilot studies. |
| Mikasa, K. 2016 | In cases of severe ventilator-associated pneumonia (VAP), the selection of broad-spectrum antimicrobial drugs or combination therapy with them should be used without delay. | Strong recommendation | I: Randomized comparative study | —— |
| Kalil, A.C. 2016 | In patients with suspected VAP, the inclusion of coverage for S. aureus, Pseudomonas aeruginosa, and other gram-negative bacilli is recommended in all empiric regimens. | Strong recommendation | Low-quality evidence | Observational studies; Retrospective review; RCTs; Meta-analyses of RCTs; Meta-analyses of observational studies; Meta-analyses of diagnostic studies; Rs /meta-analyses developed by the guideline panels |
|  | The inclusion of an agent active against MRSA is only suggested for the empiric treatment of suspected VAP in patients with any of the following: a risk factor for antimicrobial resistance, patients being treated in units where >10%–20% of S. aureus isolates are methicillin resistant, and patients in units where the prevalence of MRSA is not known. | Weak recommendation | Very low-quality evidence |  |
|  | The inclusion of an agent active against methicillin sensitive S. aureus (MSSA) (and not MRSA) is suggested for the empiric treatment of suspected VAP in patients without risk factors for antimicrobial resistance, who are being treated in ICUs where <10%–20% of S. aureus isolates are methicillin resistant. | Weak recommendation | Very low-quality evidence |  |
|  | If empiric coverage for MRSA is indicated, either vancomycin or linezolid is recommended. | Strong recommendation | Moderate-quality evidence |  |
|  | When empiric treatment that includes coverage for MSSA (and not MRSA) is indicated, a regimen including piperacillin-tazobactam, cefepime, levofloxacin, imipenem, or meropenem is suggested. | Weak recommendation | Very low-quality evidence |  |
|  | The use of 2 antipseudomonal antibiotics from different classes is only suggested for the empiric treatment of suspected VAP in patients with any of the following: a risk factor for antimicrobial resistance, patients in units where＞10% of gram-negative isolates are resistant to an agent being considered for monotherapy, and patients in an ICU where local antimicrobial susceptibility rates are not available. | Weak recommendation | Low-quality evidence |  |
|  | The use of one antibiotic active against P. aeruginosa is suggested for the empiric treatment of suspected VAP in patients without risk factors for antimicrobial resistance who are being treated in ICUs where≤10% of gram-negative isolates are resistant to the agent being considered for monotherapy. | Weak recommendation | Low-quality evidence |  |
|  | Avoiding aminoglycosides in patients with suspected VAP is suggested if alternative agents with adequate gram-negative activity are available. | Weak recommendation | Low-quality evidence |  |
|  | Avoiding colistin in patients with suspected VAP is suggested if alternative agents with adequate gram-negative activity are available. | Weak recommendation | Very low-quality evidence |  |
|  | For patients with VAP due to gram-negative bacilli that are susceptible to only aminoglycosides or polymyxins (colistin or polymyxin B), both inhaled and systemic antibiotics are suggested, rather than systemic antibiotics alone. | Weak recommendation | Very low-quality evidence | SRs /meta-analyses of RCTs; SRs / meta-analyses of observational studies; Rs/meta-analyses developed by the guideline panels. |
|  | For patients with VAP, a 7-day course of antimicrobial therapy is recommended rather than a longer duration. | Strong recommendation | Moderate-quality evidence |  |
|  | Treatment of MRSA HAP/VAP with either vancomycin or linezolid rather than other antibiotics or antibiotic combinations is recommended. | Strong recommendation | Moderate-quality evidence | Observational studies; meta-analysis; RCTs; |
|  | For patients with HAP/VAP due to P. aeruginosa, the choice of an antibiotic for definitive (not empiric) therapy based upon the results of antimicrobial susceptibility testing is recommended. | Strong recommendation | Low-quality evidence | Observational studies; meta-analysis; RCTs. |
|  | There is a strong recommendation against aminoglycoside monotherapy for patients with HAP/VAP due to P. aeruginosa. | Strong recommendation | Very low-quality evidence | Observational studies; meta-analysis; RCTs. |
|  | For patients with HAP/VAP due to P. aeruginosa who are not in septic shock or at a high risk for death, and for whom the results of antibiotic susceptibility testing are known, monotherapy using an antibiotic to which the isolate is susceptible rather than combination therapy is recommended. | Strong recommendation | Low-quality evidence | Observational studies; meta-analysis; RCTs. |
|  | For patients with HAP/VAP due to P. aeruginosa who remain in septic shock or at a high risk for death when the results of antibiotic susceptibility testing are known, combination therapy using 2 antibiotics to which the isolate is susceptible rather than monotherapy is suggested. | Weak recommendation | Very low-quality evidence | Observational studies; meta-analysis; RCTs. |
|  | For patients with HAP/VAP due to P. aeruginosa, there is a strong recommendation against aminoglycoside monotherapy. | Strong recommendation | Very low-quality evidence | Observational studies; meta-analysis; RCTs. |
|  | For patients with HAP/VAP due to ESBL-producing gram-negative bacilli, the choice of an antibiotic for definitive (not empiric) therapy be based upon the results of antimicrobial susceptibility testing and patient-specific factors is recommended. | Strong recommendation | Very low-quality evidence | Observational studies; meta-analysis; RCTs. |
|  | In patients with HAP/VAP caused by Acinetobacter species, treatment with either a carbapenem or ampicillin/ sulbactam if the isolate is susceptible to these agents is suggested. | Weak recommendation | Low-quality evidence | Observational studies; meta-analysis; RCTs. |
|  | In patients with HAP/VAP caused by Acinetobacter species that is sensitive only to polymyxins, intravenous polymyxin (colistin or polymyxin B) is recommended and adjunctive inhaled colistin is also suggested. | Weak recommendation | Low-quality evidence | Observational studies; meta-analysis; RCTs. |
|  | In patients with HAP/VAP caused by Acinetobacter species that is sensitive only to colistin, the use of adjunctive rifampicin is not suggested. | Weak recommendation | Moderate-quality evidence | Observational studies; meta-analysis; RCTs. |
|  | In patients with HAP/VAP caused by Acinetobacter species, there is a recommendation against the use of tigecycline. | Strong recommendation | Low-quality evidence | Observational studies; meta-analysis; RCTs. |
|  | In patients with HAP/VAP caused by a carbapenem-resistant pathogen that is sensitive only to polymyxins, intravenous polymyxins (colistin or polymyxin B) is suggested. | Weak recommendation | Moderate-quality evidence | Observational studies; meta-analysis; RCTs. |
|  | In patients with HAP/VAP caused by a carbapenem-resistant pathogen that is sensitive only to polymyxins, adjunctive inhaled colistin is suggested. | Weak recommendation | Low-quality evidence | Observational studies; meta-analysis; RCTs. |
|  | For patients with HAP/VAP, it is recommended that antibiotic therapy be de-escalated rather than fixed. | Weak recommendation | Very low-quality evidence | Observational studies; meta-analysis; RCTs. |
| Mehta, Y. 2014 | —— | —— | —— | —— |
| Klompas, M. 2014 | —— | —— | —— | —— |
| Alvarez-Lerma, F. 2014 | —— | —— | —— | —— |
| Li, YM 2013 | It is recommended that patients with VAP should be treated empirically as soon as possible. | Strong recommendation | Low quality of evidence | Prospective observation study |
|  | The initial empirical anti-infective treatment of VAP patients is usually recommended to be single drug anti-infective treatment with appropriate antibacterial spectrum; If the pathogen is multi drug resistant, the combination treatment of antibiotics can be selected. | Strong recommendation | Moderate quality of evidence | Meta-analyses of RCTs; RCTs |
|  | For pulmonary infection caused by multidrug-resistant non fermenting bacteria, when the effect of systemic anti infection treatment is poor, the combination of aerosol inhalation of aminoglycosides or polymyxin and another drug is recommended. | Strong recommendation | Low quality of evidence | RCTs; Review; consensus summary. |
|  | VAP anti infection course is generally recommended as 7-10 days. If this has poor clinical efficacy or the patient has, multi drug resistant bacterial infection or immune function defect, the treatment time can be appropriately prolonged. | Strong recommendation | Moderate quality of evidence | RCTs |
|  | It is recommended that anti-infective therapy for VAP patients uses a step-down strategy. | Strong recommendation | Low quality of evidence | Observational studies |
| Gupta, D. 2012 | It is recommended that every ICU/hospital should have its own antibiotic policy for initiating empiric antibiotic therapy in HAP based on their local microbiological flora and resistance profiles. | Strong recommendation | High-quality evidence | —— |
|  | In hospitals that do not have their own antibiotic policy, the policy given in these guidelines is recommended. | Strong recommendation | Low quality of evidence | —— |
|  | Although there is no evidence to suggest that combination therapy is superior to monotherapy, the expert group recommended initial empiric therapy as a combination due to the high prevalence rates of MDR pathogens in late-onset HAP/VAP and with an aim to ensure the chances of appropriateness of the initial regimen (UPP). However, once the culture reports are available, the regimen should be de-escalated to the appropriate monotherapy. | Strong recommendation | High-quality evidence | Guidelines; Consensus document; meta-analysis of RCTs; RCTs; observational, multicenter, cohort studies. |
|  | In patients with VAP due to Pseudomonas, Acinetobacter, and MRSA, a longer duration (14 days) of antibiotic course is recommended. | Strong recommendation | High-quality evidence | Prospective observative studies; RCTs; Review; Guidelines. |
|  | In other patients with VAP who are clinically improving, a 7-day course of antibiotics is recommended. | Strong recommendation | High-quality evidence | Prospective observative studies; RCTs; Review; Guidelines. |
|  | Aerosolized antibiotics (colistin and tobramycin) may be a useful adjunct to intravenous antibiotics in the treatment of MDR pathogens where toxicity is a concern. | Strong recommendation | Moderate quality of evidence | Meta-analysis; cohort studies; RCTs; prospective study; retrospective studies; case series. |
|  | It is recommended that aerosolized antibiotics should not be used as monotherapy and should be used concomitantly with intravenous antibiotics. | Strong recommendation | Moderate quality of evidence | Meta-analysis; cohort studies; RCTs; prospective study; retrospective studies; case series. |

HAP: Hospital-acquired Pneumonia; VAP: Ventilator-associated Pneumonia; ICU: Intensive Care Unit; MDR: Multidrug resistance; MRSA: Methicillin-resistant Staphylococcus aureus; MSSA: Methicillin sensitive Staphylococcus aureus; SR: Systematic Review; RCT: Randomized Controlled Trial.
